# Leptin, insulin and thyroid hormones in a cohort of Egyptian obese Down syndrome children: a comparative study

**DOI:** 10.1186/1472-6823-12-22

**Published:** 2012-10-15

**Authors:** Sohier Yahia, Reham M EL-farahaty, Amany K El-Hawary, Mona A El-hussiny, Hanaa Abdel-maseih, Faeza El-Dahtory, Abdel-Hady El-Gilany

**Affiliations:** 1 Department of Pediatrics, Faculty of Medicine, Mansoura University Children Hospital, Mansoura, Egypt; 2 Department of Clinical Pathology, Faculty of Medicine, Mansoura University, Mansoura, Egypt; 3 Cytogenetic Lab, Genetic Unit, Children Hospital, Mansoura University Children Hospital, Mansoura, Egypt; 4 Department of Public Health, Faculty of Medicine, Mansoura University, Mansoura, Egypt

**Keywords:** Down syndrome, Obesity, Leptin, Leptin resistance, Insulin resistance

## Abstract

**Background:**

Obesity is a major worldwide health problem. It is commonly observed in Down syndrome individuals than in the general population. The reason for increased risk of obesity in DS is unclear.

The current study was designed to clarify differences in some obesity- related hormones in a group of prepubertal Down syndrome children.

**Methods:**

Thirty six Egyptian children with Down syndrome were enrolled in this study, divided according to their body mass index (BMI) into 23 obese and13 non obese. Another group of 43 non Down children were recruited, they were divided according to their BMI into 20 patients having simple obesity and 23 non obese, as control groups. Fasting blood samples were collected for estimation of fasting blood glucose (FBG), insulin, leptin, free thyroxin (FT_4_), thyroid stimulating hormones (TSH) and creatine kinase (CK). Insulin resistance was assessed by Homeostasis Model Assessment method (HOMA-IR). The ratio of leptin to BMI (LEP/BMI) was used as an index of leptin resistance.

**Results:**

Median values of FBG, insulin, and HOMA-IR were significantly higher in Down versus non Down groups, while median values of leptin and leptin resistance were non-significantly different among Down versus non Down groups. Median TSH values were non- significantly different between obese Down and obese non Down. Although the median values of TSH and FT4 were within normal range in Down groups, four cases of subclinical hypothyroidism were encountered. Leptin levels were correlated with insulin and IR but not with TSH in Down groups.

**Conclusion:**

Increased circulating leptin, a marker of leptin resistance in obese children with Down syndrome seems to be similar to that in children with simple obesity. Elevated FBG and insulin in obese Down children highlights the presence of early IR. Associated myopathy evidenced by mildly elevated CK levels could be an added factor for obesity in such group of patients.

## Background

Down syndrome (DS, trisomy 21) is the most common chromosomal abnormality affecting live-born infants. Children with DS demonstrate an increased risk of developing various endocrine disorders, such as hypothyroidism, diabetes mellitus and childhood obesity [1]. Unique characteristics of this syndrome such as reduced resting metabolic rates [2] or decreased physical activity [3] and increased risk of diabetes or hypothyroidism [4] may play a role in the development of obesity. In addition, children with DS may have a tendency to overeat, since the cerebral regions that are responsible for weight regulation may be damaged [5,6] moreover, there is incapacity to do long physical exercise in DS individuals because the effort can be quickly perceived as painful, difficult and without sense for them, which contributes to adoption of a sedentary lifestyle [7].

Obesity is known to increase the risk of morbidity and mortality in DS persons [8]. Obesity is characterized by an increase of adipose tissue mass [9]. Adipocytes secrete leptin hormone, which acts in the hypothalamus to suppress appetite and control body weight [10]. In addition, insulin regulates energy homeostasis through control of appetite and energy expenditure [11]. Both hormones rise in direct proportion to adipose mass, they cross blood brain barrier and have receptors in the arcuate nucleus [12].

Although leptin production is positively correlated with the percentage of body fat, it is postulated that obese individuals, with hyperleptinemia, have some degree of leptin resistance [10]. It is unclear whether this mechanism is similar in children with DS.

### The aim of the work

The current study was designed to clarify differences in some obesity- related hormones in a group of prepubertal Down syndrome children.

## Methods

The present study was conducted on two groups: group I, comprised 36 children having DS confirmed as trisomy 21 by chromosomal study their age ranged from 2 to 10 years. All were living within their families and were of same socioeconomic class, they were not assigned for special diet regimen. They were recruited from outpatient clinic of Pediatric Genetic Unit, Mansoura University Children Hospital; they were enrolled in physical and speech therapy programs to help their motor skills and language development. They were divided according to BMI into; non obese Down (NOD) group of 13 cases, of whom 7 males and 6 females with BMI percentiles > 5^th^ to ≤ 85^th^, their ages ranged from 2 to 8 years, and the obese Down (OD) group of 23 cases, of whom 13 males and 10 females, their age ranged from 2.5 to 10 years, with BMI percentiles > 95^th^. Group II comprised 43 children, without DS to act as a control group. They were divided according to BMI into; 20 obese non Down patients (OND) including 12 males and 8 females to represent simple obesity they were recruited from Endocrinology unit, Mansoura University Children Hospital. Their age ranged from 2.8 to 11 years, and 23 non obese non Down (NOND) healthy children including 12 males and 11 females, their age ranged from 2 to 10 years. The study was approved by the research ethics committee of Mansoura University. All groups are subjected to thorough history and clinical examination including the presence of acanthosis nigricans. Anthropometric data were collected as part of an ongoing research program ascertaining standing height to the nearest 0.1cm in bare feet using portable Harpender stadiometer and weight to the nearest 0.1kg using digital weight scale, the measurements were repeated twice and the average was recorded. BMI was calculated for groups as weight (kg) divided by height (m) squared [13]. We excluded children who had started pubertal development according to Tanner and Whitehouse staging (1976) [14] to avoid hormonal changes induced by puberty. Patients having chronic conditions affecting growth or energy balance and children who already were taking thyroxine, corticosteroids or immunosuppressive drugs were excluded.

Laboratory data: Blood samples were collected after a-12 hours overnight fast for estimation of fasting blood glucose (FBG), insulin, leptin, Thyroid stimulating hormone (TSH), Free thyroxin (FT_4_) and creatine kinase (CK). FBG was estimated by liquizyme GOD- PAP supplied by Human, Germany [15]; serum insulin by chemilumeinescent immunometric assay using IMMULITE® 1000 supplied by Siemenes Healthcare Diagnostics, USA [16], serum leptin by enzyme-linked immunosorbent assay (ELISA) supplied by DRG GmbH, Germany [10] and serum CK levels using CK-NAC liquid, UV kinetic method (Human,Germany) [15]; and serum TSH and FT_4_ concentrations by electrochemiluminescence immunoassay (ECLIA) using Elecsys 2010, Roch Diagnostics GmbH, Germany [17]. Insulin resistance (IR) was calculated according to homeostasis model assessment-estimated insulin resistance (HOMA-IR), through the formula: FBG (mmol/L) x fasting insulin (μIU/ml)/22.5 [18]. The ratio of leptin to BMI (LEP/BMI) was used as an index of leptin resistance (Leptin- R). This index measures leptin levels while controlling for the contribution of BMI [19].

### Statistical analysis

Data were analyzed using SPSS statistical package version 16. Categorical variable (sex) was presented as number and percent. Chi square test was used for comparison between groups. Numerical variables were tested for normality by Kolmogorov-Smirnov test and found to be non parametric in distribution. So Kruskal Wallis was used for comparison between four groups while, Mann Whitney was used for comparison between two groups. Spearman's correlation coefficient was used to test the correlation between variables within each group of patients. P≤0.05 was considered to be statistically significant.

## Results

Table 1 showed comparison between studied groups regarding age, sex, FBG, obesity- related hormones and CK. Regarding age and sex there were no significant difference between four groups (P= 0.160, 0. 962).

**Table 1:** Comparison between studied groups regarding age, sex, FBG, some obesity- related hormones and CK

**Parameters**	**Groups**				
	**OD**	**NOD**	**OND**	**NOND**	**P**^**#**^
	**n=23**	**n=13**	**n=20**	**n=23**	
**Age (years)**					
Median	5	4	6	5	0.160
Range	2.5-10	2-8	2.8-11	2-10	
**Sex**					
Male	13(56%)	7(53%)	12(60%)	12(52%)	0.962
Female	10(43%)	6(46%)	8(40%)	11(48%)	
**FBG** (mmo/l)					
Median	6.2^ab^	5.2 ^ac^	5.6 ^b^	4.6 ^c^	<0.001
Range	5.2-6.8	4.4-6.3	2.7-6.10	4-5.2	
**Insulin** (uU/ml)					
Median	20 ^ab^	7 ^ac^	10.9 ^b^	3.8 ^c^	<0.001
Range	9-56	4-10	4.6-18.5	2-6	
**HOMA-IR**					
Median	5.8 ^ab^	1.56 ^ac^	2.58 ^b^	0.77 ^c^	<0.001
Range	2.2-8.35	0.9-2.57	0.77-4.88	0.38-1.38	
**Leptin** (ng/ml)					
Median	18 ^a^	3 ^a^	18.25	3	<0.001
Range	11-36	1.8-10	10.4-45.7	1.9-7.0	
**leptin R**					
Median	0.68 ^a^	0.21 ^a^	0.67	0.195	<0.001
Range	0.4-1.26	0.13-0.46	0.45-1.2	0.146-0.304	
**TSH** (uU/ml)					
Median	3	2.8 ^c^	3.3	1.3 ^c^	<0.001
Range	0.85-7.30	1-5.7	0.7-2.7	0.4-3.0	
**FT4** (pmol/L)					
Median	16 ^b^	16.2	12 ^b^	14	<0.001
Range	12-22	13-20	10.4-14.8	12.5-15	
**CK** (U/L)					
Median	60 ^b^	50 ^c^	29 ^b^	30 ^c^	<0.001
Range	12-193	35-130	18-47	24-50	

OD: Obese Down. NOD: Non obese Down.

OND: Obese non Down. NOND: Non obese non Down.

#: Kruskal wallis test was used except for association with sex, Chi square test was used.

a: Significant P_(MW)_<0.05, comparing OD Vs NOD groups.

b: Significant P_(MW)_<0.05, comparing OD Vs OND groups.

c: Significant P_(MW)_<0.05, comparing NOD Vs NOND groups.

Considering Down groups; OD group showed significantly higher median values of FBG, insulin, HOMA-IR, leptin and leptin-R compared with NOD group (P< 0.001), while no significant differences as regard TSH, FT_4_ and CK.

Considering the two obese groups, OD group showed significantly higher median values of FBG, insulin, HOMA-IR, FT_4_ and CK compared with OND group (p< 0.001). The increase in median values of FT4 and CK were within reference range, but no significant differences as regard leptin and leptin-R between both groups. At the same time, among the two normal BMI groups (>5to ≤85 percentiles), NOD group showed significantly higher but still within reference range median values of FBG, insulin, HOMA-IR, TSH, FT_4_ and CK compared with NOND group (P = 0.003, <0.001), while no significant differences as regard leptin and leptin-R were present between both groups.

Table 2 showed correlations between studied parameters in each group. Significant positive correlations were observed between age and each of leptin, leptin-R and insulin in both OD and NOND groups and only between age and leptin in OND group. Also significant positive correlation were observed between leptin and each of insulin, HOMA-IR and leptin resistance in OD, NOD and NOND groups, while in OND group significant positive correlation were observed only between leptin and leptin –R, but no correlation were found between leptin and TSH . As regard CK there were significant positive correlation with TSH in both OD and NOND groups.

**Table 2:** Correlations between some studied parameters in each group

**Parameters**	**OD**		**NOD**		**OND**		**NOND**	
	**r**	**P**	**R**	**p**	**r**	**p**	**r**	**p**
**Age:**								
and leptin	0.913	**<0.001**	0.557	**0**.**048**	0.510	**0.021**	0.947	**<0.001**
and leptin -R	0.925	**<0.001**	0.089	0.772	0.440	0.052	0.792	**<0.001**
and Insulin	0.575	**0.004**	0.305	0.310	0.310	0.183	0.795	**<0.001**
**Leptin:**								
and insulin	0.619	**0.002**	0.722	**0.005**	0.336	0.148	0.775	**<0.001**
and HOMA-IR	0.608	**0.002**	0.626	**0.022**	0.402	0.079	0.806	**<0.001**
and leptin- R	0.969	**<0.001**	0.709	**0.007**	0.985	**<0.001**	0.899	**<0.001**
and TSH	0.170	0.438	0.472	0.104	0.222	0.347	0.645	**0.001**
**CK and TSH**	0.636	**0.001**	0.194	0.524	0.371	0.108	0.433	**0.039**

## Discussion

The main findings of the present study are the association between hyperinsulinemia, impaired fasting glucose and obesity in obese Down children, that could make them at higher risk to develop IRS. Also there is elevation of leptin and leptin-R median values in both OD and NOD groups with normal thyroid profile in down children. However four cases of subclinical hypothyroidism associated with CK above 90 U/L values were observed. This study was one of the few to report median and range values of some hormones related to obesity in a cohort of Egyptian DS obese and non obese prepubertal children.

### IR in children with DS

One of the characteristics of the person with trisomy 21 is the propensity for becoming obese [2]. IR is the most common metabolic alteration related to obesity [20]. Considering the upper limit of FBG up to 5.55 mmol/L [21], normal fasting range for insulin varies from 5 to 15 μIU/ml [22] and fasting insulin greater than 15μIU/ml indicated IR [12]. This study showed significant higher median values of FBG, insulin and IR (assisted by HOMA-IR) in Down (OD, NOD) versus non Down (OND, NOND) groups with highest median values in OD group. This could be attributed to high levels of adipose tissue (which is more central than peripheral) in such children [22,23] and the fact that central adiposity is more associated with IR [22]. Magge et al. [24] observed significantly higher percent body fat, non significantly higher insulin and glucose in children with DS than unaffected siblings, while no significant difference in HOMA-IR. It was suggested previously in small size study, (15 cases, including one prepubertal child with DS) that increased HOMA-IR and high rate of glucose production indicated presence of peripheral and hepatic IR in adolescent with DS [22].

Considering one of the two definitions of IR syndrome in children: [1] the presence of any three or more of hyperinsulinemia, overweight, high systolic blood pressure, high triglycerides, low HDL-C and impaired fasting glucose; and [2] the presence of hyperinsulinemia and at least two of the other five risk factors [25]. Our study reported an association between hyperinsulinemia, impaired fasting glucose and obesity in obese Down children, that could make them at higher risk to develop severe metabolic complications. In contrary Flore et al., 2008 [26] could not display IR in a group of young non obese adults with DS.

### Leptin, leptin-R & obesity in DS children

Considering leptin and leptin-R, a higher median value of leptin in OD children was observed in this study, however, no significant difference in leptin and leptin -R were observed between OD versus OND, indicating an increase in median leptin values, a marker of leptin resistance in obese children irrespective to the cause of obesity. This was in contrary to the suggestion of presence of genetic basis for obesity (three copies of chromosome 21) that might be a cause of more severe leptin resistance in DS [24]. Previously Considine et al. [10] concluded that obese individuals have higher leptin levels than normal weight individuals, and obesity is associated with leptin resistance. This belief is in harmony with our results that showed positive correlation between leptin and leptin-R in obese groups (OD, OND).

### Insulin leptin interrelationship

Consistent with insulin/leptin arcuate nucleus of the hypothalamus axis, insulin and glucose appear to stimulate leptin secretion in adipocytes. In response, leptin decreases insulin secretion via direct action on leptin receptors in pancreatic B-cells while enhancing their actions and consequently improves whole body insulin sensitivity [27].

So, obesity promoted - hyperinsulinemia stimulates leptin release. Like other biological signaling pathways, leptin appears to regulate its own receptor signaling or increased central leptin results in reduced hypothalamic leptin receptor expression and leptin signaling. Hence, obesity promotes hyperleptinemia, which in turn self promotes leptin resistance and further obesity, making leptin resistance both a consequence and cause of obesity [27].

### Thyroid dysfunction in DS

Thyroid dysfunction is highly prevalent in DS persons. The clinical symptoms and signs of both DS and hypothyroidism are overlapping to some extent e.g. hypotonia, mental retardation, growth failure, macroglosia, obesity etc. [28]. The presence of undetected hypothyroidism in children with DS could compound the problems in already compromised situation.

We observed significantly higher FT_4_ but not exceeding reference range (1–5 years: 11.9 – 22.1; 6–10 years: 11.5-19.96pmol/L) [29] with no significant difference in TSH values in Down versus non Down groups. This was in contrary to previous data reported by Konings et al. [4] and Shaw et al. [28] who found higher mean TSH values (6.5±1.3, 5.4±2.9mU/l respectively) in face of normal FT_4_ or T_4_. They hypothesized that thyroid gland in children with DS needs an elevated TSH pressure to produce sufficient hormones [4]. However, we observed elevated serum TSH values with normal thyroxine levels in four cases, three in OD and one in NOD groups denoting subclinical hypothyroidism, associated with CK above 90U/l [30].

### Hypotonia, CK & associated myopathy in DS children

Considering hypotonia as a risk factor for obesity in DS, we suggest that it could be associated with muscle breakdown. CK is considered the primary marker for muscle breakdown, in response to exercise, a portion of the population has exaggerated increases in CK [31,32]. Reasons for an exaggerated response are not fully understood but may reflect genetics [31], fiber type, an underlying myopathy, and/or some other environmental behavioral factors, including fitness level [33]. Furthermore variations in muscle mass have been proposed to account for interracial differences in total CK activity [34]. Our results showed significantly higher median CK value in OD compared with OND group. Also a positive correlation was found between CK and TSH in OD group. Associated myopathy either endocrine [35] (hypothyroid related myopathy) or infectious secondary to viral infection [36], strenuous physical activity [32] may be the underlying causes for these mildly elevated CK values and muscle weakness could be added to the hypotonia which is a part of genetic predisposition of the disease.

## Conclusions

Increased circulating leptin, a marker of leptin resistance in obese Down children seems to be similar to that in children with simple obesity, this could point to an absence of genetic basis for more leptin resistance in such high risk population for obesity. The association between fasting hyperglycemia and hyperinsulinemia in obese children with DS indicates early IR, suggesting a possible genetic basis. Associated myopathy could be an added factor for obesity as proved by the mildly elevated CK values. Large sample size, studying other causes of DS and further genetic study are needed to prove the genetic role in this tendency for obesity in DS.

### Limitations

The study was small in size, single center, cross sectional comparative study. It relied solely on BMI for age percentiles of Center of Disease Control and Prevention as an indicator of body fatness because it is simple, easily obtained than skin folds thickness, presence of standardized BMI curves and cut-off points defining overweight & adiposity in childhood [37] and correlates well with DEXA [38]. Previous results, suggest that skin fold measurements may only slightly improve (8%) the estimation of body fatness among children who are obese (BMI ≥95th CDC percentile) [39]. Furthermore it may help determine if an overweight child may have excess body fatness [39] and this group of children were not included in our study. Finally due to racial variation and poor inter- and intra- observer reliability skin fold thickness equations should be validated in each population and it is difficult to standardize [39]. While for waist circumference, no internationally accepted cut points for either the classification of obesity or presence of increased metabolic risk in children as in adult [25], furthermore, no data on population with DS can be found on this regard [40]. Body fat itself is not necessarily a precise measure of health risk. As stated by the recent AMA expert committee [41], “High levels of body fat are associated with increasing health risks. However, no single body fat value, whether measured as fat mass or as percentage of body weight, clearly distinguishes health from disease or risk of disease”.

## Competing interests

The authors declare that they have no competing interests.

## Authors’ contributions

SY conducted and draft the study, participated in its design and coordination and shared in clinical diagnosis and recruiting of patients. RME carried out laboratory studies participated in study design, shared in writing the manuscript and data acquisition. AKE participated in clinical diagnosis and recruiting of patients, shared in collection of data, drafting and reviewing the study. MAE and HA helped performing laboratory studies. FE performed the chromosomal study of patients. AHE performed statistical analysis. All authors read and approved the final manuscript.

## Pre-publication history

The pre-publication history for this paper can be accessed here:

http://www.biomedcentral.com/1472-6823/12/22/prepub

## References

[B1] Grammatikopoulou MG Manai A Tsigga M Tsiligiroglou-Fachantidou A Galli-Tsinopoulou A Zakas A Nutrient intake and anthropometry in children and adolescents with Down syndrome - a preliminary study Dev Neurorehabil 2008 11 4 260 7 10.1080/17518420802525526 19031198

[B2] Murray J Ryan-Krause P Obesity in children with Down syndrome: background and recommendations for management Pediatr Nurs 2010 36 6 314 9 21291048

[B3] Whitt-Glover MC O'Neill KL Stettler N Physical activity patterns in children with and without Down syndrome Pediatr Rehabil 2006 9 2 158 64 16449075 10.1080/13638490500353202

[B4] Konings CH Van Trotsenburg AS Ris-Stalpers C Vulsma T Wiedijk BM De Vijider JJ Plasma thyrotropin bioactivity in Down syndrome children with subclinical hypothyroidism Euro J Endocrinol 2001 144 1 4 10.1530/eje.0.1440001 11174830

[B5] Luke A Sutton M Schoeller DA Roizen NJ Nutrient intake and obesity in prepubescent children with Down syndrome J Am Diet Assoc 1996 96 1262 7 10.1016/S0002-8223(96)00330-6 8948387

[B6] Reinehr T Dobe M Winkel K Schaefer A Hoffmann D Obesity in Disabled Children and Adolescents An Overlooked Group of Patients Dtsch Arztebl Int 2010 107 15 268 75 20458368 10.3238/arztebl.2010.0268 PMC2864441

[B7] Bricout VA Dey S Endocrine and autonomic nervous adaptations during physical exercise in Down syndrome Genetics and etiology of Down syndrome 2011 Tech publisher 236 Available from: http://www.intechopen.com/books/genetics-and-etiology-of-down syndrome/endocrine-and-autonomic-nervous-adaptations-during-physical-exercise-in-down-syndrome, 2011

[B8] Melville CA Cooper SA McGrother CW Thorp CF Collacott R Obesity in adults with Down syndrome: a case–control study Intellectual Disability Res J 2005 49 2 125 33 10.1111/j.1365-2788.2004.00616.x 15634321

[B9] Cernkovich ER Deng J Bond MC Combs TP Harp JP Adipose-specific disruption of signal transducer and activator of transcription 3 increases body weight and adiposity Endocrinology 2008 149 4 1581 90 10.1210/en.2007-1148 18096662 PMC2276706

[B10] Considine RV Sinha MK Heiman ML Kriauciunas A Stephens TW Nyce MR Serum immunoreactive leptin .concentrations in normal-weight and obese humans N Engl J Med 1996 334 292 5 10.1056/NEJM199602013340503 8532024

[B11] Lopaschuk GD Ussher JR Jaswal JS Targeting intermediary metabolism in the hypothalamus as a mechanism to regulate appetite Pharmacol Rev 2010 62 237 264 10.1124/pr.109.002428 20392806

[B12] Ten S Maclaren N Insulin Resistance Syndrome in children J Clin Endocrinol Metab 2004 89 6 2526 39 10.1210/jc.2004-0276 15181020

[B13] Myrelid A Gustafsson J Ollars B Annerén G Growth charts for Down's syndrome from birth to 18 years of age Arch Dis Child 2002 87 97 103 10.1136/adc.87.2.97 12138052 PMC1719180

[B14] Tanner JM Whitehouse RH Clinical longitudinal standards for height, weight, height velocity, weight velocity, and stages of puberty Arch Dis Child 1976 51 170 9 10.1136/adc.51.3.170 952550 PMC1545912

[B15] Tietz NW Clinical guide to laboratory tests 1995 3 Philadelphia: WB Saunders 268 73

[B16] Reeves WG Insulin antibody determination: theoretical and practical considerations Diabetologia 1983 24 399 403 6350077 10.1007/BF00257336

[B17] Nicoloff JT Spencer CA The use and misuse of the sensitive thyrotropin assays J Clin Endoc Metab 1990 71 553 58 10.1210/jcem-71-3-553 2203796

[B18] Matthews DR Hosker JP Rudenski AS Naylor BA Treacher DF Turner RC Homeostasis model assessment:insulin resistance and β-cell function from fasting blood glucose and insulin concentrations in man Diabetologia 1985 28 412 19 10.1007/BF00280883 3899825

[B19] Lee JH Reed DR Price RA Leptin resistance is associated with extreme obesity and aggregates in families Int J Obes 2001 25 1471 3 10.1038/sj.ijo.0801736 11673768

[B20] Chiarelli F Marcovecchio ML Insulin resistance and obesity in childhood Eurpean Journal of Endocrinology 2008 159 s67 s74 10.1530/EJE-08-0245 18805916

[B21] American Diabetes Association Diagnosis and classification of Diabetes mellitus Diabetes Care 2011 34 s62 s69 21193628 10.2337/dc11-S062 PMC3006051

[B22] Fonseca CT Amaral DM Ribeiro MG Beserra IC Guimaraes MM Insulin resistance in adolescents with Down syndrome: a cross-sectional study BMC Endocr Disord 2005 5 1 6 10.1186/1472-6823-5-1 15963228 PMC1177940

[B23] Bare MT Waldron J Gumm H Van Dyke DC Chang H Van Dyke DC, Lang DJ, Heide F, Van Duyne S, Soucek MJ Nutrition assessment of the child with Down syndrome Clinical perspectives in the management of Down syndrome 1990 USA: Springer-Verlag New York Inc 107 25

[B24] Magge SN O'Neill KL Shults J Stallings VA Stettler N Leptin levels among prepubertal children with Down syndrome compared with their siblings J Pediatr 2008 152 3 321 6 10.1016/j.jpeds.2007.08.008 18280834 PMC2267910

[B25] Steinbeck KS Insulin resistance syndrome in children and adolescent: clinical meaning and indication for action Int J Obesity 2004 28 829 32 10.1038/sj.ijo.0802723 15208648

[B26] Flore P Bricout VA Van Biesen D Guinot M Laporte F Pépin JL Eberhard Y Favre-Juvin A Wuyam B van de Vliet P Faure P Oxidative stress and metabolism at rest and during exercise in persons with Down syndrome Eur J Cardiovasc Prev Rehabil 2008 15 35 42 10.1097/HJR.0b013e3282f2bff3 18277183

[B27] Martin SS Qasim A Reilly MP Leptin resistance. A possible interface of inflammation and metabolism in obesity-related cardiovascular disease JACC 2008 52 1201 10 18926322 10.1016/j.jacc.2008.05.060 PMC4556270

[B28] Shaw CK Thapalial A Nanda S Shaw P Thyroid dysfunction in Down syndrome KUMJ 2006 4 2 182 6 18603895

[B29] Kapelari K Kirchlechner C Högler W Schweitzer K Virgolini I Moncayo R Pediatric reference intervals for thyroid hormone levels from birth to adulthood: a retrospective study BMC Endocr Disord 2008 8 15 10.1186/1472-6823-8-15 19036169 PMC2645400

[B30] Kupke IR Tritschler W Kather B Bablk W Creatine kinase NAC activated reference values for children Klin Padiator 1980 192 4 348 50 10.1055/s-2008-1035606 7192770

[B31] Clarkson PM Hoffman EP Zambraski E Gordish-Dressman H Kearns A Hubal M ACTN3 and MLCK genotype associations with exertional muscle damage J Appl Physiol 2005 99 564 9 10.1152/japplphysiol.00130.2005 15817725

[B32] Totsuka M Nakaji S Suzuki K Sugawara K Sato K Break point of serum creatine kinase release after endurance exercise J Appl Physiol 2002 93 1280 6 12235026 10.1152/japplphysiol.01270.2001

[B33] Ebbeling CB Clarkson PM Exercise-induced muscle damage and adaptation Sports Med 1989 7 207 34 10.2165/00007256-198907040-00001 2657962

[B34] Gledhill RF Van der merwe CA Greyling TM Van Niekerkt MM Race-gender differences in serum creatine kinase activity: a study among South Africans J of Neurology, Neurosurgery, and Psychiatry 1988 51 301 304 10.1136/jnnp.51.2.301 PMC1031551 3346700

[B35] Pavlu J Carey MP Winer JB Hypothyroidism and nemaline myopathy in an adult J Neurol Neurosurg Psychiatry 2006 77 708 9 10.1136/jnnp.2005.079293 16614046 PMC2117463

[B36] Kumar K Guirgis M Zieroth S Lo E Menkis AH Arora RC Freed DH Influenza myocarditis and myositis: case presentation and review of the literature Can J Cardiol 2011 27 514 22 10.1016/j.cjca.2011.03.005 21652168

[B37] Al Husain M Body mass index for Saudi children with Down’s syndrome Acta Paediatric 2003 92 1482 5 10.1111/j.1651-2227.2003.tb00836.x 14971803

[B38] Lobstein T Baur L Uauy R Obesity in children and young people: a crisis in public health Obes Rev 2004 5 4 10 10.1111/j.1467-789X.2004.00133.x 15096099

[B39] Freedman DS Sherry B The Validity of BMI as an indicator of body fatness and risk among children Pediatrics 2009 124 S 1 523 34 10.1542/peds.2008-3586E 19720664

[B40] González-Agüero A Ara I Moreno LA Vicente-Rodríguez G Casajús JA Fat and lean masses in youths with Down syndrome: gender differences Res Dev Disabil 2011 32 5 1685 93 10.1016/j.ridd.2011.02.023 21435834

[B41] Barlow SE Expert committee recommendations regarding the prevention, assessment, and treatment of child and adolescent overweight and obesity: summary report Pediatrics 2007 120 s164 s92 10.1542/peds.2007-2329C 18055651

